# Can Responsible Ownership Practices Influence Hunting Behavior of Owned Cats?: Results from a Survey of Cat Owners in Chile

**DOI:** 10.3390/ani9100745

**Published:** 2019-09-29

**Authors:** Sebastián Escobar-Aguirre, Raúl A. Alegría-Morán, Javiera Calderón-Amor, Tamara A. Tadich

**Affiliations:** 1 Departamento de Ciencias Animales, Facultad de Agronomía e Ingeniería Forestal, Pontificia Universidad Católica de Chile, Santiago 8940000, Chile; sebastian.escobar@uc.cl; 2 Fundación Ciencia Ciudadana, Providencia, Santiago 7500000, Chile; 3 Departamento de Medicina Preventiva Animal, Facultad de Ciencias Veterinarias y Pecuarias. Universidad de Chile, Santiago 8820808, Chile; ralegria@veterinaria.uchile.cl; 4 Facultad de Ciencias Agropecuarias, Universidad Pedro de Valdivia, Santiago 7500908, Chile; 5 Instituto de Ciencia Animal, Facultad de Ciencias Veterinarias, Universidad Austral de Chile, Isla Teja, Valdivia 5090000, Chile; j.calderon.amor@gmail.com; 6 Departamento de Fomento de la Producción Animal, Facultad de Ciencias Veterinarias y Pecuarias, Universidad de Chile, Santa Rosa 11735, La Pintana, Santiago 8820808, Chile

**Keywords:** owned cat, responsible ownership, wildlife, prey, *Felis catus*, hunting behavior

## Abstract

**Simple Summary:**

Cats are popular pets, with increases in both the number of households that have cats and the number of cats per household. Cats can also have great negative impacts on wildlife. In Chile, the potential influence of owned cats on wildlife has not been studied, which is why the aim of this study was to investigate the number and type of prey that cats bring home and its relationship with responsible ownership practices. For this, we sent a questionnaire to pet owners across the country. The survey included questions about the type of household and pets, if cats had brought prey back home, and responsible ownership practices. The results showed that from 5216 respondents, 94.3% had a pet; from these, 49.9% had at least one cat. There were on average 2.2 cats per household, and 84.1% of owners reported that their cats had brought back prey. Birds and mammals were the most common type of prey, followed by insects. The lack of responsible ownership practices such as not being registered, not having a litter box, having free access to the outdoors, or living in a house with a garden and providing a hiding place increased the probability of cats bringing back home prey.

**Abstract:**

The domestic cat (*Felis catus*) has become a worldwide threat to wildlife. The potential impact of owned cats on wildlife in Chile has not been documented at a large scale. The purpose of this study was to investigate the number and type of prey that owned cats bring back in Chile and its relation with responsible ownership practices. An online survey was distributed to 5216 households that included questions about the type of pet, responsible ownership practices, and in the case of cats, the type of prey they brought home. Descriptive statistics as well as univariate and multivariate logistic regression analysis were applied. The results showed that 94.3% of respondents had a pet, and from these, 49.9% had at least one cat. A total of 84.1% of owners reported that their cats had brought back prey. Birds were the most common type of prey, followed by mammals and insects. Not being registered with a microchip, not having a litter box, living in a house with access to a garden, not having a hiding place for the cats, and having free access to the outdoors significantly increased the odds of cats bringing back prey. Body condition score or providing ad libitum food to cats did not have an effect on bringing prey.

## 1. Introduction

There is historical and paleontological evidence suggesting that wildcats were domesticated as early as 10,000 years ago [1,2]. Historically, cats were domesticated and kept near the household because of their ability to hunt, and thus were kept as pest control agents [3]. Nowadays, there are around 600 million cats around the world [4], and many of them are considered as companion animals and family members [1,4,5]. Human population dynamics have a direct impact on the composition of the population of domestic animals with which they live [6]. In the USA, it is described that there are more than 74 million pet cats with an average of 2.1 cats per cat-owning household [4], while in the UK, it is estimated that 25% of the population has a cat [7]. In Chile, 36% of households have only dogs, 16% have cats and dogs, and 11% have only cats, with a total of 64% of households having at least one pet [8].

Cat management depends on the perception of cats by society. A large number of owners report that cats have some level of outdoor access [5,9]. This leads to the existence of a considerable number of free-roaming animals. The term “free-roaming” refers to the lack of confinement, encompassing animals with an owner or strays (lost or abandoned) [10]. There are demographic factors and entrenched beliefs that contribute to fostering the practice of allowing cats to have outdoor access. For example, the general belief that cats are more independent and that they do not need as much care as dogs can encourage owners to allow cats to roam freely [9,11]. The consequences of this practice have effects at different levels. Free-roaming domestic cats are associated with a greater risk of being bitten by another cat [9] and contracting diseases [12], among other negative effects on their welfare. It is also considered a public health issue [13,14]. Moreover, a topic of considerable scientific and social debate is the indirect and direct effects of cats on wildlife [15,16].

There is extensive evidence that shows the high impact that cats have on wildlife [17,18,19]. Cats predate a wide variety of wild animals such as invertebrates [20,21], birds, and mammals [19,22,23]. Although cats that are provided adequate food have lower predation rates than those that are poorly fed [17], domestic cats maintain their hunting behavior. Baker et al. [19] reported a predation rate of 21 prey/cat/annum, while Lepczyk et al. [23] found a predation rate between 0.8–1.4 birds killed/cat/week. In the USA, 3–4 billion birds and 6–22 billion mammals are killed annually by free-roaming domestic cats [24]. Together with the direct impact of predation, are the indirect effects such as disease transmission and the “fear effect” that cats inflict on prey populations [18].

The negative ecological impact is well recognized. In the meta-analysis carried out by Doherty et al. [25], cats were linked with the extinction of 75 species, mostly birds. Lepczyk et al. [23] found that among birds preyed by cats, there were species of conservation concern. Medina and García [21] reported endemic invertebrate species preyed on by cats in Canary Island. In southern Chile, a study carried out by Silva-Rodríguez and Sieving [17] showed that cats preyed on several endemic and threatened mammals. 

One method to evaluate the rate of predation is to ask the owners the number and type of prey that the animals return home. This number only represents a portion of prey items. Loyd et al. [22] monitored with video cameras the outdoor activity of cats in Georgia (USA), and found that only 23% of captures were brought home, and the rest were abandoned (49%) or eaten (28%). Kays and DeWan reported on average 1.67 prey brought home per cat per month in Albany, New York, USA [26].

Considering the continued expansion of urban areas and the lack of regulation, the impact of cat predation as an ecological threat is potentially increasing in Chile. Today, there are no published accounts of the impact of owned cats in Chile. This is why the aim of this study was to investigate through a questionnaire for cat owners, the types of prey species returned home by their cats and associate it with responsible ownership practices.

## 2. Materials and Methods

A questionnaire was constructed using the Google Forms tool in order to assess cat owners’ responsible ownership practices. The questionnaire consisted of three sections, and only accepted one response per user. Section one asked for the demographic information of participants (gender, age, region of residence, type of house); the second section contained three questions to assess how informed owners were in relation to national legislation associated to animal welfare and the responsible ownership of pets; finally, the third section addressed 11 management practices associated with cats and two questions regarding whether cats brought back prey and what type of prey. 

For the two questions that addressed the hunting behavior of cats, these were formulated as follows. (1) Has your cat brought back home prey as a present for you? (2) What type of prey? In question (1), hunting behavior was associated with cats “bringing a present” in order to avoid sensitivity and social desirability [27]. Thus, if hunting behavior is perceived as negative in the community, saying that their cat has indulged in this behavior and answering “yes” could be perceived as likely to be judged as an undesirable behavior (sensitivity), likewise to avoid owners answering “no” in order to seek approval (social desirability). All questions were closed except the last one, which was an open question where owners could write down the type of prey. No personal information was requested, and all participants had to provide an informed consent before starting the questionnaire. The estimated sampling size for each macrozone was determined a priori assuming 95% power at an alpha of 0.05. The data from the last national demographic (INE) survey was used for the total number of households per macrozone [28]; a sample size of 385 surveys per macrozone was estimated.

Survey participants were recruited through social networks such as Facebook, Instagram, and Twitter; the survey was also distributed through mailing lists. The questionnaire was open between June and August of 2018 (Chilean winter). 

After closing the form, data was downloaded into an Excel spreadsheet and frequencies, means, standard deviations, and percentages were calculated. Considering the nature of the collected information (retrieving prey behavior of cats, registered as a binary or dichotomous response) a logistic regression model analysis was performed, where Y (response) can have only two values, 0 or 1 (Y = 0 or Y = 1), representing cats that do retrieve (1) and those that do not retrieve (0) any type of prey [29]. A univariate logistic regression analysis was performed to assess the relationship between the retrieving-prey behavior of cats (retrieves or does not retrieve), and the responsible ownership practices assessed through the questionnaire. Variables with a *p*-value ≤ 0.15 were selected for the multivariable logistic regression model; a stepwise backward elimination procedure was carried out, using the log likelihood ratio test (LRT), the model with the lowest LRT was selected as the final model, variables whose regression coefficients were not significant (*p* > 0.05) were removed from the multivariable logistic regression [30]. Non-significant variables, which produced a change greater than 20% in the regression coefficients of the significant variables when removed, were retained in the model in order to adjust for confounding factors, and all the biologically feasible interaction terms were included in the model building [29]. The goodness-of-fit of the final model was evaluated using the Hosmer and Lemeshow test [31]. Biologically logical interaction between variables that fullfill the liberal criterial were also analyzed. All categorical variables were analyzed by constructing dummy variables approach [32]. 

The analysis was carried out using RStudio and the statistical software R [33], plus ‘lme4’, ‘ggplot2’, and ‘gcookbook’ packages. The odds ratio, 95% confidence interval, and *p*-value were computed.

## 3. Results

**Figure 1:**
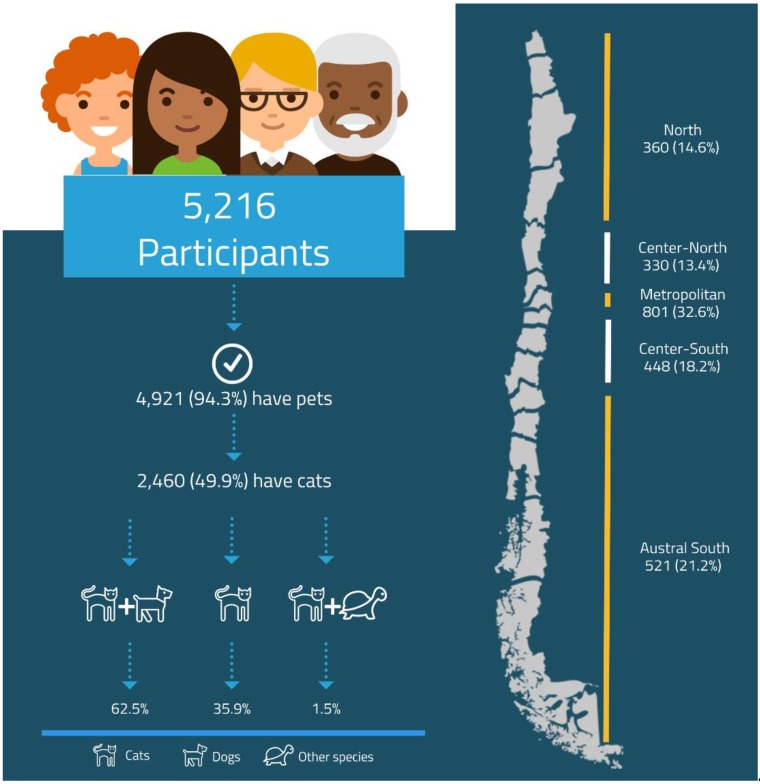
Distribution of participants (n = 2460) that declare having cats only or with other animals as pets, and distribution of cat owners according to macrozone of Chile.

A total of 5216 participants (households) responded to the questionnaire; from this, 4921 declared to have one or more types of pets. Within the pet owners group, 49.9% have at least one cat, and the majority of cat owners also have a second species as a pet (69%) (Figure 1). A total of 2460 households have cats, with the number of cats per household varying between 1–24, with a mean of 2.2 ± 2 cats per household and a total number of 5438 cats informed in this study.

According to the demographic characteristics of the survey, 90.3% of respondents were female, 8.9% were male, and 0.8% preferred not to say. The age of participants varied between 18–80 years of age (mean = 35.9 ± 13.2 years). Most cat owners who answered the survey lived in houses with a garden (76.8%) and were concentrated in the Metropolitan Macrozone (32.6%), while the lowest number of questionnaires retrieved came from the central North Macrozone (Figure 1). 

**Figure 2:**
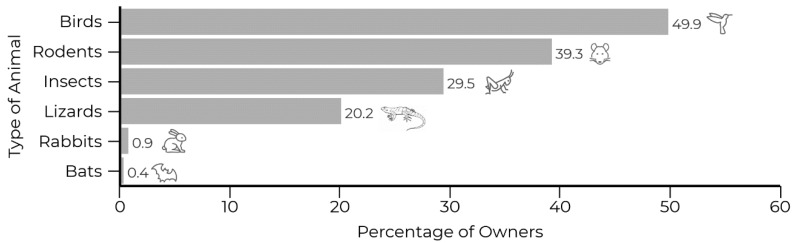
Percentage of owners that declare that their cats have brought back home a prey according to the type of animal.

Most owners (84.1%) report that their cat has brought at least one prey home (Figure 2), birds being the most common type of prey (49.9%), followed by rodents (39.3%), insects (29.5%), lizards (20.2%), rabbits (0.9%), and bats (0.4%) (Figure 2). 

In relation to knowledge of the Chilean legislation, most owners declare to feel very informed or informed about the Chilean Animal Protection Law and the Chilean Responsible Pet Ownership Law (74.5% and 89.3% respectively). Less than half of respondents (40.2%) declare to be informed about the five domains of animal welfare (five freedoms). 

The univariate analysis showed that almost all the variables fulfilled the criteria established for inclusion in the multivariate model (Table 1). According to the multivariate logistic regression analysis, cats that were not registered with a microchip had 1.402 times more probability of bringing back home prey; cats that were kept in apartments with garden access had 1.682 times more probability of bringing back home prey. The interaction between living in a house with no access to garden and not having access to the outdoors shows that those cats had 1.822 times more probability of bringing back home prey. On the other hand, owners that provided a litter box for each cat had a reduction of almost 50% in the probabilities of cat bringing back home prey; owners that did not provide free access to the outdoors had a reduction of around 57% in the probabilities of cat bringing back home prey. A similar result was obtained for owners that did not provide access to a hiding site in the house, decreasing in almost 43% the probabilities of cat bringing back home prey. Finally, owners who kept cats in houses with no garden had a decrease of around 37% in the probabilities of cat bringing back home prey (Table 2). The model presented a good fit to the data, as evidenced by the Hosmer–Lemeshow test (*p* = 0.26).

## 4. Discussion

This is, to our knowledge, the first questionnaire applied to a large sample of pet owners in order to understand the impact that responsible ownership practices can have over cats retrieving prey in Chile. Other studies that have been done to understand pet ownership in Chile are those questionnaires applied by Growth For Knowledge-Chile (GFK) [8], where 4800 households were surveyed face to face. The GFK study has a commercial objective and only contains information on whether households had a pet or not, the type of pet, macrozone, the amount of money spent on pets, if owners take them to the veterinarian, and if owners are interested in animal protection. No questions about more specific responsible ownership practices or if cats retrieved prey to the household are included [8].

In relation to the type and number of pets, our results are in agreement with the GFK questionnaire, where dogs are still the most common pet in Chile, followed by cats and other species. The percentage of households with pets in our study reached 94.3%, which is much higher than the 64% reported by the GFK survey [8], or the 68% of households with pets reported for the United States of America [34]. This difference could be associated to the sampling method used, the present study applied an online survey, which has the limitation that people that have pets might be keener to respond to it, while the GFK questionnaire used a randomized sampling method, visiting the selected households [8]. Web surveys are a simple and economic means of getting access to large samples, but it has to be considered that two types of bias occur. First there is under-coverage, since it can only cover a section of the population that has internet access [35]. In the case of Chile, internet penetration has reached 87% of households according to the last internet access survey applied by the Ministry of Transport and Telecommunications in 2017. Secondly, self-selection bias occurs instead of probability sampling, where respondents are those that have internet access, decide to open the survey from the website, and then decide to participate. Thus, the researcher is not in control of the selection process [35]. On the other hand, web surveys have the advantage of decreasing social desirability, leading to more honest responses due to the absence of an interviewer [36]. This is an important point, considering that people could feel to be judged negatively if their cats had brought back home prey. However, it should be considered that a potential bias in this study could have occurred if owners consider “bringing back home prey as a present” as a positive behavior. Distribution according to macrozone and age of respondents was similar to the GFK questionnaire, in which the main difference was the gender of respondents. In the present study, most of the respondents were females. Gender differences in the use of online resources have been described: Women are more likely to participate in internet activities that are characterized by communication and information exchange, such as online surveys [37]. 

In terms of awareness of the Chilean Animal Protection [38] and Responsible Companion Animal Ownership [39] laws, most respondents feel that they are “very informed” or “informed”; these percentages are much higher than those reported by the 2018 PDSA Animal Well-Being Report from the United Kingdom [7], where over one-third of participants were not aware of the animal welfare acts. On the other hand, UK citizens are more familiar with the five needs of the animal welfare concept than Chilean participants. Although most respondents declare to be aware of the Chilean laws, the percentage of registered cats was low (28.6%), which is a practice that is mandatory, and 65.9% of cats are allowed free access outdoors, which is a practice that is forbidden, according to the responsible ownership law [39]. Nevertheless, the number of spayed individuals and animals with their vaccines and deworming up to date was high, which are practices that are also mandatory according to law. 

The cat population has increased in Europe and the United States in the last few years. In addition to new owners, a problem that has arisen is that existing owners acquire new cats [7,34,40]; however, reliable figures of cat population in Latin America are difficult to find. In the present study, most cats are in multi-cat households (50.3%), with a mean of 2.2 cats per household, and 65.9% have free access to the outdoors. This is similar to reports from the UK and France, where multi-cat households are characterized by individuals sharing an area that includes access to the outdoors [41]. There is increasing evidence of the negative impacts that domestic cats can have on biodiversity [18,25,42], being considered as one of the most harmful 100 invasive species worldwide [43]. Despite this, in a recent study, a survey was applied to veterinary students in Chile, and only 32.7% considered that cats could have a negative impact on native species, although 78% recognized that control programs for cats should exist at the national level [44]. The effect of multi-cat households on cats’ welfare is controversial. Some studies report that it can be detrimental for cats’ welfare, since they are forced to co-exist with other individuals, resulting in a potential source of feline stress [45] and environment where intercat aggression occurs, especially the during introduction of new cats [46]. However, other authors have not found an effect on fecal glucocorticoid metabolites [47] nor on basal urinary cortisol levels [48]. The effects of multi-cat households on cat’s welfare may depend on the kinship of cats, since littermates present more affiliative behaviors than unrelated cats [49].

The application of questionnaires to understand the effect of cats on wildlife and estimate predation rates has been widely used [19,23,42]. According to our questionnaire, the majority of owners declare that their cats have brought back home prey (84.1%). Cats that do not bring prey home might not be killing wildlife at all, or killing, but not bringing it back home [50]. It has to be considered that only one-third or half of prey is returned home [24,26]. Predation rates vary according to the literature, from 0.58 [19] to 6.57 [51] prey items per cat per month. The number of prey brought home represents only part of their hunting activity [22], thus representing the minimum number of animals killed by month. Still, the impact that cats have on wildlife does not appear to strike the news or be a societal concern as much as dogs’ impact on animals and the economic losses associated with their attacks to farm animals in the country [52]. This could be the result of people perceiving bringing prey back home as “bringing a present” [53], and as a consequence of this, having a better bond with their pet. At the same time, historically, cats have been kept both as a house pet and a pest control agent [3]; thus, hunting behavior could be seen as a positive quality.

Hunting is a natural behavior in cats; they are solitary specialized hunters, and their ability to hunt is one of the reasons of why they were domesticated in the first place [45,53]. In relation to the type of prey, the most common one was birds; this is in accordance with Schuttler et al. [54], who also found that birds were the most affected species by cat predation at the Cape Horn Biosphere Reserve in the south of Chile (*n* = 27 cats). Dickman and Newsome [55] and Loss et al. [24] also reported that cats had a higher predation rate on birds, contrary to unowned cats, who showed a higher predation rate on mammals. This could be associated to the higher availability of birds in the household gardens and urban areas than mammals. Birds were followed by mice and rats, and then insects and lizards (Figure 2), similar to the findings of Woods et al. [50]. Invertebrates such as insects are also under a high predation pressure by cats; this could be because they are smaller and more abundant [20]. According to Eisenhauer [20], the impact of cat predation on invertebrates should be monitored in order to understand its effect on biodiversity and related functions and services [20]. In the study of Medina and García [21] in the Canary Islands, insects were the most common invertebrate prey. The authors also highlight that the identification of insect species is essential to understand the significance of cat predation—for example, if endangered insect species are being affected [21].

In relation to the responsible ownership practices, we did not find a relationship with the body condition score of cats, nor having or not ad libitum access to food with bringing back a prey. This is similar to the findings of Dickman and Newsome [55], where owners reported that their cats are well fed and have access to ad libitum food and still hunt live prey. Owners following appropriate feeding strategies for their cats will not necessarily reduce their motivation to hunt. The hunting behavior sequence includes capture, killing, and consuming components, which are relatively independent of each other, resulting in that hunger does not need to be present in order to motivate the performance of the first two components [56]. Keeping cats indoors reduces the probability of hunting, as shown by the results of the univariate analysis and multivariate logistic regression. This would especially be true in the case of birds, mammals, and reptiles, but not necessarily in the case of insects; many owners that declare to have their cats indoors did mention that their cats hunt moths and other insects that enter the household. Woods et al. [50] indicated that keeping cats indoors at night could reduce their access to wild mammals that are predominantly nocturnal. Other strategies that have been implemented to reduce cat attacks include equipping them with bells that can warn the prey about their presence [50].

Not being registered with a microchip and not providing a litter box for the cat inside the household resulted in practices that increased the probability of cats bringing back prey. Not having a microchip could possibly be associated with owners not practicing other responsible ownership practices that could directly affect hunting behavior, such as allowing free access to the outdoors. Households with cats should have at least one litter box per cat located in different areas of the house, as an insufficient number or the nonexistence of them may induce welfare problems such as feline idiopathic cystitis or stress [53]. Not providing a litter box can also promote cats to select an alternative toileting site, usually near their hiding sites [53]. Therefore, not providing a litter box and having free access outdoors could be encouraging cats to go outside for this resource and thus facilitating hunting behavior. The interaction of not providing free access to the outdoors and living in a house without a garden could encourage cats to explore further spaces, without owner’s supervision in order to find a place to recreate and objects for enrichment. This would be of special importance for predation on insects that could get inside the house more easily. The provision of environmental enrichment would be beneficial for these cats. The fact that providing a hiding place within the house increased the odds of retrieving prey is contrary to expectations, since it would be logical to think that cats without a hiding place would seek for this resource outside, and thus increase its chances of hunting. One plausible explanation could be that cats that are provided with a hiding space have lower stress scores [57,58], and thus are more fit to hunt. A second possible alternative is that hiding places are complex areas within their comfort space, and cats are more attracted to hunt in open areas that differ from their surroundings [56], increasing the chances of hunting from 17% in dense grass or complex areas up to 70% in open areas [59].

Factors such as the sex or age of cats were not included in this study, but prior studies have not found evidence that these influence hunting behavior [22,54].

## 5. Conclusions

This is the first report for Chile on the implications of some responsible ownership practices over the predation behavior of urban-owned cats at a large scale. Our results are in line with most reports worldwide that demonstrate the potential damaging effect of domestic cats on wildlife, mainly birds and mammals, but also on insects. It is also important to highlight that responsible ownership practices such as allowing cats free access outdoors, providing litter boxes, and providing hiding places can have a significant effect on cats bringing back home prey. As in other studies, food provision and body condition score did not influence hunting behavior, since capture and killing behavior are not necessarily triggered by hunger. Education strategies are required at the national level to sensitize cat owners about their responsibilities and the negative effects that cats can have on wildlife. 

## Figures and Tables

**Table 1:** Description of responses provided by cat owners (n = 2460) according to household and responsible ownership characteristics (number and percentage), and their association with cats bringing home any type of prey established through univariate logistic regression analysis. The *p*-value is provided.

Variable	Categories	N	*p*-Value
Vaccines up to date	Yes	1570	reference
	No	735	<0.001
	Not informed	155	0.012
Deworming up to date	Yes	1932	reference
	No	415	0.148
	Not informed	148	0.097
Registered with microchip	Yes	705	reference
	No	1749	<0.001
Access to a litter box	No	602	reference
	1 per cat	1368	<0.001
	<1 per cat	479	<0.001
Free access to the outdoors	Yes	1620	reference
	No	830	<0.001
Access to a hiding site in the house	Yes	2291	reference
	No	165	<0.001
House with garden	Yes	1889	reference
	No	571	<0.001
Apartment without garden	Yes	401	reference
	No	2059	<0.001
Macrozone	North	360	reference
	Center-North	330	0.896
	Metropolitan	801	0.827
	Center-South	448	0.129
	Austral-South	521	0.116
Awareness of animal protection law	Very aware	441	reference
	Aware	1393	<0.001
	Not aware	461	0.016
	Not sure	165	0.102
Awareness of responsible ownership law	Very aware	659	reference
	Aware	1538	0.091
	Not aware	206	0.802
	Not sure	57	0.709
Awareness of five domains of animal welfare	Very aware	432	reference
	Aware	556	0.624
	Not aware	1178	0.272
	Not sure	294	0.627
BCS (body condition score)	1 or 2	526	reference
	3	1005	0.211
	4 or 5	929	0.039
Cats are spayed	All	1947	reference
	Just female	191	0.181
	Just male	60	0.057
	No	259	0.399
Access to food	Controled	1672	reference
	Ad libitum	786	0.063

**Table 2:** Final model results from the multivariate logistic regression analysis. The odds ratio, 95% confidence interval (C.I.), lower and upper limit, and *p*-value are reported for the variables that were retained in the model and had an association with cats bringing back home prey.

Variable	Categories	Odds Ratio (OR)	95% CI		*p*-Value
			Lower Limit	Upper Limit	
**(Intercept)**	-	-	-	-	<0.001
Registered with microchip	Yes				
	No	1.402	1.107	1.776	0.005
Access to a litter box	No				
	1 per cat	0.554	0.382	0.803	0.002
	<1 per cat	0.67	0.44	1.021	0.062
Free access to the outdoors	Yes				
	No	0.438	0.325	0.592	<0.001
Access to a hiding site in the house	Yes				
	No	0.572	0.393	0.832	0.003
House with garden	Yes				
	No	0.613	0.377	0.996	0.048
Apartment with garden	No				
	Yes	1.682	1.067	2.651	0.025
Free access to the outdoors: House with garden	Yes:Yes				
	No:No	1.822	1.095	3.032	0.021
